# Worth Remembering

**Published:** 1891-09

**Authors:** 


### WORTH REMEMBERING.

The juice of half a lemon in a teacup of strong black coffee, without sugar, will often cure a sick headache.

The skin of a boiled egg is the best remedy for a boil. Carefully peel it, wet and apply to the boil ; it draws out the matter and relieves soreness.

For simple hoarseness take a fresh egg, beat it and thicken with pulverized sugar. Eat freely of it and the hoarseness will soon be relieved,

When your face and ears burn so terribly bathe them in very hot water—as hot as you can bear. This will be more apt to cool them than any cold application.

Castor oil may be comfortably taken in hot milk, in a half-wineglass of weak punch in hot water sweetened and highly flavored with essence of peppermint or wintergreen.

A sure cure for inflammatory rheumatism is made by taking one ounce pulverized saltpetre and putting it into a pint of sweet oil. Bathe the parts affected and a sound cure will speedily be made.

Neuralgia in the face has been cured by applying a mustard plaster to the elbow. For neuralgia in the head, apply the plaster to the back of the neck. The reason for this is that mustard is said to touch the nerves the moment it begins to draw or burn, and to be of most use must be applied to the nerve centers, or directly over the place where it will touch the affected nerve most quickly.

The custom, in spite of modern sanitary teaching, still prevails of keeping the occupant of a sick-room at all hours in a darkened room. There is no sense in this; it is as if the attendants were anticipating the death of the patient; and, if the reason is asked for, it is as inconsistent as the act. The reason usually offered is that the patient cannot bear the light—as though the light could not be cut off from the patient by a curtain or screen, aad as though to darken one part of the room it were necessary to darken the whole of it. The real reason is an old superstitious practice connected with small-pox and other terrible diseases involving the exclusion of light. A more injurious practice really could not be maintained, as by it a great remedy is lost. Sunlight diffuses through a room and warms and clarifies the air. It has a direct influence on the minute organic poisons—a distinctive influence that is most precious—and it has a cheerful effect upon the mind. The sick should never be gloomy, and, in the presence of the light the shadows of gloom fly away. Happily this fact is now recognized in hospital practice, and should be equally so in private practice.

